# Suture-Mediated Patent Foramen Ovale Closure Using the NobleStitch EL: Results from a Hospital-Based HTA

**DOI:** 10.3390/ijerph19137863

**Published:** 2022-06-27

**Authors:** Giovanni Gaetti, Alessandro Beneduce, Dario La Fauci, Alessandro Scardoni, Federica Chiappa, Lorenzo Bellini, Michela Franzin, Anna Maria Natale, Paola Marras, Paolo Ranieri, Carlo Signorelli, Eleonora Bossi, Lucrezia Ferrario, Emanuela Foglia, Matteo Montorfano, Anna Odone

**Affiliations:** 1 School of Public Health, Vita-Salute San Raffaele University, 20132 Milan, Italy; gaetti.giovanni@hsr.it (G.G.); scardoni.alessandro@hsr.it (A.S.); chiappa.federica@hsr.it (F.C.); bellini.lorenzo@hsr.it (L.B.); bossi.eleonora@hsr.it (E.B.); 2 Interventional Cardiology Unit, Cardio-Thoracic-Vascular Department, IRCCS San Raffaele Scientific Institute, 20132 Milan, Italy; beneduce.alessandro@hsr.it (A.B.); montorfano.matteo@hsr.it (M.M.); 3 HTA Committee, IRCCS San Raffaele Hospital, 20132 Milan, Italy; lafauci.dario@hsr.it (D.L.F.); franzin.michela@hsr.it (M.F.); natale.annamaria@hsr.it (A.M.N.); marras.paola@hsr.it (P.M.); ranieri.paolo@hsr.it (P.R.); signorelli.carlo@hsr.it (C.S.); 4 Centre for Research on Health Economics, Social and Health Care Management, Carlo Cattaneo-LIUC University, 21053 Castellanza, Italy; lferrario@liuc.it (L.F.); efoglia@liuc.it (E.F.); 5 Department of Public Health, Experimental and Forensic Medicine, University of Pavia, 27100 Pavia, Italy

**Keywords:** Health Technology Assessment, AdHopHTA, hospital-based HTA, PFO, systematic review

## Abstract

(1) Background: Patent foramen ovale (PFO) is a congenital abnormality present in up to 25% of the general population, and it is a relevant cause of cryptogenic stroke. We applied the hospital-based HTA model (AdHopHTA) to conduct a multidimensional assessment of NobleStitch EL, an innovative suture-mediated PFO closure device. We compared it to Amplatzer PFO Occluder (APO) to provide evidence to inform technologies’ governance in hospital settings. (2) Methods: For each AdHopHTA dimension we: systematically retrieved available evidence from the literature applying the PRISMA guidelines and then analyzed original clinical and cost data of a NobleStitch EL device at San Raffaele research hospital in Milan (Italy). The economic dimension was analyzed through activity-based costing and a cost analysis. We conducted semi-structured interviews with selected healthcare professionals to explore the organizational, legal, social, and ethical impact. (3) Results: A single study was included for the NobleStitch EL, with 10 for APO. Both literature data and original data showed comparable safety. Efficacy data analysis found that the PFO closure was at 89% for NobleStitch EL vs. 89–97% for APO. APO has a better impact on the budget and minor process costs. Consulted experts reported that the organizational impact of NobleStitch EL in the short and the long run as null, albeit a better impact under the social and the ethical aspects. (4) Conclusion: We suggest that there is inadequate evidence to conclude the relative efficacy of NobleStitch EL as compared to APO. Nevertheless, this report shows a good safety profile and higher costs for NobleStitch EL, with no organizational or legal impact. Further studies in selected population are recommended.

## 1. Introduction

The IRCCS San Raffaele Hospital (HSR) in Milan is one of the largest teaching hospitals in Northern Italy, with a well-structured surgical department comprising an Interventional Cardiology Unit, with special expertise in the field of patent foramen ovale (PFO) closure. The heart anatomical defect called PFO is a common condition present in up to 25% of the general population, and it might cause severe diseases such as cryptogenic stroke. As better explained later, in some patients, the PFO may become symptomatic. Thus, to prevent any relapse, they might go under treatment. Internationally, the current standard of care for PFO closure is using the percutaneous, metallic, double-disc Amplatzer PFO Occluder (APO), which overcame the medical therapy and other devices used in the last decades [1]. Table S1 in the Supplementary Material provides an overview of randomized clinical trials of percutaneous PFO closure. NobleStitch EL was introduced as an innovative suture-mediated PFO closure device, consisting of a percutaneous suture stitch of the heart defect without any permanent device implant. Based on the encouraging results reported in the ongoing clinical study at HSR, the Interventional Cardiology Unit staff proposed to the hospital Health Technology Assessment (HTA) Commission to carry out a full technology assessment to provide a multi-dimension evaluation of the NobleStitch EL device [2,3].

To produce a full, hospital-based HTA report, a multidisciplinary team, composed of interventional cardiologists, clinical engineers, procurement and operations experts, a pharmacist expert in ethical evaluations, and public health medical doctors expert in HTA methodology, was established. In detail, we aimed to answer the following research question: “From a hospital-based point of view, is NobleStitch EL a suitable alternative to Amplatzer PFO Occluder in patients with symptomatic PFO undergoing closure?”

## 2. Materials and Methods

We applied the hospital-based HTA model (AdHopHTA), assuming a San Raffaele Hospital perspective, comparing the NobleStitch EL system to the gold standard APO, examining both technologies under the following domains included in the AdHopHTA framework: health problem and current use (D1); description and technical characteristics (D2); safety aspects (D3); clinical effectiveness (D4); cost and economic evaluation (D5); and ethical (D6), organizational (D7), social (D8), legal (D9), and political and strategic aspects (D10).

This analysis was conducted with original clinical data on NobleStitch EL and systematic reviews of the literature for both devices since no original data was available on APO. Original economic data and experts’ opinions were collected for both devices. Details on the data sources used in the multidimensional evaluation for each dimension are reported in Table 1.

### 2.1. Original Clinical Data Analysis

The original data were based on 134 patients with symptomatic PFO evaluated for the treatment with NobleStitch EL between July 2017 and August 2019 at San Raffaele Hospital. The clinical study focused on safety and on efficacy endpoints. The safety endpoints were in-hospital and follow-up all-cause death, device, and procedure-related adverse events. The efficacy endpoint was a 3-month effective PFO closure evaluated by trans-esophageal echocardiography (TEE) follow-up, defined as residual right-to-left shunt (RLS) grade ≤ 1 at microbubbles test. Secondary efficacy endpoints were procedural success, (defined as the correct delivery of both septal sutures followed by negative intraprocedural contrast injection and/or trans-thoracic echocardiography (TTE) or TEE microbubbles test) and complete PFO closure, defined as RLS grade 0 at 3-month TEE follow-up [4].

All patients undergoing percutaneous, suture-mediated PFO closure with the NobleStitch EL device at San Raffaele Hospital between July 2017 and August 2019 were prospectively included in this study. Patients were considered eligible for percutaneous PFO closure if they had experienced a documented cryptogenic cerebrovascular accident, including ischemic stroke or transient ischemic attack (TIA); complained of intractable migraine; were exposed to the potential risk of decompression sickness due to professional reasons (professional scuba diving); or were scheduled for neurosurgical procedures requiring prolonged sitting with anesthesia and positive-pressure invasive ventilation and had evidence of PFO with atrial RLS.

Data regarding procedure technical details and in-hospital outcomes were prospectively collected from medical records, hospital internal electronic datasets, and centralized imaging datasets. After discharge, patients were followed up by outpatient visits at 1, 3, and 6 months after the procedure. A TEE microbubbles test was scheduled for a 3-month follow-up to evaluate residual RLS.

#### Baseline Characteristics of Patients

A cohort of 134 consecutive patients was evaluated for suture-mediated PFO closure. Of them, 69 patients were excluded as shown in the Supplementary Material, and only 65 patients with suitable anatomy underwent suture-mediated PFO closure with the NobleStitch EL device. Most of the patients were female (54%) with a mean age of 48 ± 12 years. The most common indication for PFO closure was recurrent cryptogenic cerebrovascular accidents (84%), either ischemic stroke (53%) or TIA (47%), intractable migraine (12%), professional scuba diving (1.5%), and intracranial neoplasm surgery requiring sitting anesthesia and ventilation (1.5%). The microbubble test resulted positive at baseline during normal respiration in 51% of patients and 100% when Valsalva maneuver was performed. The severity of right-to-left shunt was graded: 48% of patients presented with a moderate shunt (grade 2) and 52% with a severe shunt (grade 3). No data on patients who had undergone an APO implant were collected. Of the 65 patients included in the study, 42 (65%) underwent a 3-month TEE follow-up.

Baseline clinical and demographic characteristics are shown in Supplementary Material.

### 2.2. Literature Reviews

Considering the lack of head-to-head studies in current literature, with the aim of retrieving, pooling, and critically appraising the available evidence on the intervention technology of interest and its comparator, we conducted two systematic literature reviews, following the Prepared Items for Systematic Reviews and Meta-Analysis (PRISMA) guidelines [5]. For the NobleStitch EL’s systematic review, we defined the PICOS and identified published studies by searching electronic databases: Medline, the Embase and Cochrane Library, PROSPERO, the Cochrane Central Register of Controlled Trials, the Centre for Reviews and Dissemination, the International Clinical Trials Registry platform, ClinicalTrials.gov, and the EU clinical trials register.

We included studies with NobleStitch EL as an intervention or comparator, used for patients suffering from symptomatic PFO, for any relevant outcome, and for any study design. For the APO systematic review, we defined a different PICOS and we limited our search to the MEDLINE database, including studies focusing on safety and efficacy outcomes and with a sample of at least 50 patients treated with APO, to include only studies with a significant number of patients. Complete search strategies, PRISMA flowcharts, and PICOS are available in the Supplementary Material.

Identified studies were independently reviewed for eligibility by three authors in a two-step-based process; a first screening was based on titles and abstracts, while full texts were retrieved for the second screening. Disagreements were resolved by consultation with senior authors. Data were extracted by three authors using a standardized data extraction spreadsheet for safety and efficacy endpoints.

The quality appraisal of included studies was carried out using different tools, according to study designs. Quality appraisal of included studies was carried out using the following tools (by study design): the Newcastle–Ottawa Assessment Scale for observational studies [6]; the Cochrane tool for experimental studies [7]; the Assessment of Multiple Systematic Reviews for systematic reviews and meta-analysis (AMSTAR 2) [8]; the INAHTA checklist for assessing HTA reports [9]; and the AGREE II (Appraisal of Guidelines for Research & Evaluation) for assessing guidelines [10] (Supplementary Material).

#### 2.2.1. First Systematic Review: NobleStitch EL

Details of included studies and PRISMA Flowcharts are available in Supplementary Material Figure S6. A total of 56 records were retrieved from running the search strategies on the identified databases. After removing duplicates, 53 records of primary literature, 1 original study, and 2 clinical trials were included in our review. The only original article included that used a NobleStitch EL as an intervention was published in 2018 and conducted in Italy, the UK, and Germany. It was a single-arm observational study, with a sample size of 192 patients [11], and it did not include a non-exposed group of patients. Thus, this study is considered of acceptable quality with the study design [11].

Two clinical trials with the NobleStitch EL were identified with a total of 890 patients involved. Both studies considered NobleStitch EL as an intervention, and one had APO as a comparator, the other reported no comparator. Both studies were sponsored by HeartStitch.Com. Unfortunately, both trials are still ongoing, and the results are not available. Zero studies focused on economic, organizational, social, ethical, or legal aspects.

#### 2.2.2. Second Systematic Review: APO

We identified 77 records of primary literature without duplicates. After the first and the second screenings, only 10 papers were included (see PRISMA flowchart in Supplementary Material Figure S8). Selected studies had an APO as an intervention or a comparator: 6 case-control studies, 3 cohort studies, and 1 randomized clinical trial. The sample size of the APO group ranged from 52 to 620 patients.

To validate cohort and case-control studies, the New Ottawa Scale [6] was applied. Six studies were considered of high quality (1 study 8 out 9 points [12]; 5 studies 9 out 9 points [13,14,15,16,17]). We considered the three studies conducted without a control group [18,19,20], with quality acceptable to the study design. The randomized clinical trial [1] was assessed with a Revised Cochrane risk-of-bias tool for randomized trials (RoB-2) and considered with a medium risk of bias [7]. Quality appraisal results are shown in the Supplementary Material.

### 2.3. Economic Original Data Analysis

To correctly assess the economic and the financial aspects (D5), it was necessary to analyze and to determine the costs of the clinical pathway, by focusing on the implementation of both NobleStitch EL and APO over 12 months, assuming the hospital perspective. In this view, a process mapping technique and a consequent activity-based costing (ABC) analysis were developed, according to the standard clinical pathway performed at San Raffaele Hospital.

For the economic evaluation of the process, only healthcare direct costs were considered, to measure all the costs sustained by the hospital performing these two procedures. According to the above, the following items of healthcare expenditure were accordingly valorized based on the standard consumption for each patient: (i) human resources involved; (ii) laboratory exams and diagnostic procedures; (iii) drugs; (iv) medical devices and other specific or generic disposable; (v) equipment; and (vi) general costs, such as cleaning services, hospital maintenance, energy, hospital sterilization services (defined based on the PFO length of stay). Due to confidential agreements, the price of the two devices is reported in the “drugs and disposable” field.

The economic evaluation of the two processes was integrated with a Budget Impact Analysis (BIA) comparing the baseline scenario (considering the prior standard of care in HSR, where all the patients underwent PFO closure with APO), with five different innovative scenarios (differing from higher use of NobleStitch EL in gradual substitution to APO) to define the economic sustainability of the innovative medical device. The BIA considered the hospital perspective, and it estimated the overall hospital healthcare expenditure of up to 12 months in treating 80 patients (the population meeting the inclusion criteria).

### 2.4. Expert Opinion

To explore healthcare professionals’ opinions and perceptions on safety, organizational, equity, social, ethical, and legal impacts, (D3, D6–D9), ad hoc questionnaires were administered to ten professionals with expertise in the use of both technologies (hospital managers, department heads and nurses’ coordinators—a detail of the profiles can be found in the Supplementary Material), according to a 7-item Likert scale, ranging from −3 (worst impact) to +3 (better impact).

All the items used for the deployment of each qualitative dimension are derived from the EUnetHTA Core Model Issues [21], with specific integrations and modifications, concerning the nature of the technologies being assessed. For D10, the main comments of HSR professionals were collected and the principal implications of the use of such technologies were analyzed.

## 3. Results

### 3.1. D1—Health Problem and Current Use

PFO is a widely recognized cause of cryptogenic stroke [22,23,24]. This tunnel-like space within the atrial septum arises from the separation between the septum primum and the septum secundum at the anterosuperior portion of the fossa ovalis, and it plays a significant role in fetal circulation. This anatomical communication may remain patent in up to 25% of patients, allowing passage from the venous to the systemic circulation, potentially giving rise to paradoxical embolization [22,23,24]. Similarly, based on the same mechanism, PFO has been associated with decompression sickness and hypoxemia [25,26]. This has led to the development of various devices for percutaneous PFO closure that have been largely studied and tested mainly for secondary stroke prevention [13,27].

The results of several randomized clinical trials and metanalyses, described in Supplementary Material Table S1, support the role of transcatheter PFO closure using different prosthetic, implantable occluder devices over medical therapy in preventing recurrent strokes [1,28,29,30,31,32,33,34,35,36]. This growing evidence has been recently implemented by European and International scientific societies that gave strong recommendations for percutaneous PFO closure in selected patients with cryptogenic stroke.

Scant evidence is available in the literature, concerning how many patients may need intervention for symptomatic PFO, suggesting that the yearly risk of cryptogenic stroke in healthy people with PFO may be as low as 0.1% [37].

Between July 2017 and August 2019, 163 patients were evaluated for percutaneous PFO closure at San Raffaele Hospital. Of these, 65 patients with suitable anatomy underwent NobleStitch EL implantation, 29 out of 77 in the first year of use (July 2017 to June 2018) and 36 out of 86 in the second period (July 2018 to August 2019, 14 months), registering +4% of NobleStitch EL implant (38% vs. 42%).

### 3.2. D2—Device Description and Technical Characteristics

#### 3.2.1. Intervention

The NobleStitch EL system consists of two dedicated suture delivery catheters (NobleStitch S and NobleStitch P) to capture and suture the septum secundum and the septum primum using a 4-0 polypropylene suture, which produces an “S” shaped closure of the PFO. The distal end of each NobleStitch has a suture-carrying arm that opens inside the heart to engage the septum correctly and an internal needle that pierces through the septum tissue picking the suture up in the opened suture-carrying arm. A third catheter (KwiKnot) is advanced over the septum secundum and the septum primum sutures to approximate both septa, achieving closure by securing the stitch and trimming the excess suture material. (Supplementary Material Figures S1–S3).

The procedure is performed under local anaesthesia and fluoroscopic guidance, with or without TEE monitoring. In case of device failure, the use of a second stitch or a bailout traditional PFO closure with the APO device might be permitted. Early patient mobilization and discharge are encouraged, and, at discharge, antiplatelet therapy continuation is left to the discretion of the attending physician. In the absence of other indications, the standard protocol consists of a single antiplatelet agent daily for one month [11] (Supplementary Material Figure S3).

#### 3.2.2. Comparator

The APO is the most widely used device for percutaneous PFO closure. It is a self-expandable, double-disc device made from a platinum-filled, nickel–titanium wire mesh, implanted in an endovascular technique. The right atrial desk is larger than the left atrial desk, connected with a short waist. After implantation, the device is endothelialized within 6–12 months, therefore, 1–6 months of double antiplatelet therapy is generally recommended [36].

A comparative description of the characteristics of the two devices is reported in Table 2.

### 3.3. D3—Safety

As mentioned, the safety domain was explored using the following data sources: (i) original data from clinical practice, (ii) literature data from the systematic review, and (iii) ad hoc questionnaires for expert opinion.

#### 3.3.1. Clinical Practice

Percutaneous suture-mediated PFO closure was successfully carried out in 94% of patients treated with NobleStitch EL, with correct positioning and septal sutures delivered in all cases, albeit with a significant residual shunt at intraprocedural contrast injection in four cases (final device deployment 89%). The most frequent side effect was rhythm disturbances, reported by 6 (9%) patients. Other less frequent procedural complications are shown in the Supplementary Material Figure S5.

#### 3.3.2. Systematic Literature Reviews

The mean procedure time was 58 (40–75) min for NobleStitch EL [11] and 30–40 min for APO [16,18]. Intraprocedural complications and atrial fibrillation after the procedure was reported to be 0% for NobleStitch EL, whereas for APO intraprocedural complications were between 0% and 5% [1,15,16,18] and atrial fibrillation ranged from 1.9% to 10.1% [12,14,15,16,17,19]. The device-related complications for APO were heterogeneous, ranging from 0% to 3.8% [1,12,13,16,17,18,19] and TIA was the most represented in the literature, ranging from 0% to 2.5% [1,12,16,17]. As for NobleStitch EL, the only study showed 0% for both device-related complications and TIA.

#### 3.3.3. Questionnaires

To deepen the literature evidence on the safety topic, the perceptions of healthcare professionals were retrieved. Professionals reported that both technologies are considered safe, albeit NobleStitch EL has a higher perceived safety on the “impact of associated drug therapy” (Table 3).

### 3.4. D4—Clinical Effectiveness

Several randomized clinical trials and metanalyses recommended a device-mediated percutaneous PFO closure, rather than medical therapy for secondary stroke prevention in selected patients with high-risk PFO [4]. However, in current consensus documents, the suture-mediated approach to PFO closure has not yet been considered.

#### 3.4.1. Clinical Practice

Effective PFO closure, set as effectiveness outcome, significantly improved after the procedure. Among the 65 patients treated at San Raffaele Hospital, at the 3-month TEE follow-up we observed an effective PFO closure (RLS ≤ 1) in 84% of patients, with 6 patients with RLS = 2 (14%) and 1 patient with RLS = 3 (2%).

#### 3.4.2. Systematic Literature Review

Since no head-to-head studies were published comparing NobleStitch EL and APO, we reported available data derived from the two systematic reviews of NobleStitch EL and APO separately. As for NobleStitch EL, the included study, with a follow-up of 206 days, reports a negative microbubble test (RLS = 0) in 75% of patients, measured with a 3-month TTE [11]. An effective PFO Closure (RLS ≤ 1) was found in 89% of patients, an RLS = 2 in 5.9%, and an RLS = 3 in 4.8% of patients measured with a 6-month TTE [11]. As for APO, 7 studies reported values for effective PFO closure ranging from 89% to 97% [1,13,15,16,17,18,19], measured with a 6-month TEE; and RLS ≥ 2 was seen in 3–4.5% of patients [16,18].

### 3.5. D5—Economic Financial Impact

The process mapping technique indicated that the patients, once being admitted to the Interventional Cardiology Unit with a diagnosis of “symptomatic PFO,” undergo pre-operative exams and visits: anamnesis and physical examinations, blood tests, electrocardiography, TEE with microbubble test, and the prescription of Aspirin 100 mg. The around 60-min interventional procedure is performed on the same day, by a team composed of two medical doctors, a nurse, and a radiologic technician, without the presence of an anesthesiologist (mild sedation). After the PFO procedure, the patients undergo a TEE to evaluate the device’s effectiveness, one-night hospitalization, a visit, and, without further exams, discharge. Few differences emerged between NobleStitch EL and APO in the general clinical practice and the economic impact, as they differ only in the cost of the device and few disposables, which is 1.582€ lower for APO than for NobleStitch EL (Table 3).

As for the BIA, given the methodology previously mentioned, NobleStitch EL introduction would require an additional hospital investment ranging from a minimum of 2.77% (70 APO implant and 10 NobleStitch EL implant) to a maximum of 12.47% (30 APO and 50 NobleStitch), strictly dependent on the use of the innovative medical device.

In Table 3, all the scenarios of interest for this analysis—the cumulative costs, and their impact on the budget in both absolute values and percentages—are depicted.

### 3.6. Qualitative Domains D6–D9

For most investigated items related to the ethical domain (D6), the innovative device and the comparator get similar rankings, for the safeguard of patient’s autonomy, human dignity, self-determination, and social safeguard of protected categories. NobleStitch EL is perceived slightly better in “impact on patient quality of life” related to the absence of medical therapy after the procedure as compared to at least 6 months of medical therapy after an APO implant. Patient reported outcomes were not available in the literature.

The organizational impact (D7) related to NobleStitch EL introduction in an Interventional Cardiology Unit routinely using APO is extremely limited since the only difference is related to the device. No further impact is foreseen related to hospital device acquiring activity and on departments involved in the use of the device. Mainly, the impact is related to operators’ learning curve; they are assisted for approximately 10 procedures in a training in the field, which do not engage additive resources and they do not determine a measurable quantitative impact; thus, our experts suggested a negative impact in a short-term horizon (12 months), becoming null in a long-term view (36 months).

No additional spaces, equipment, furnishings, and accessories are needed for NobleStitch EL introduction. Patients and caregivers do not need specific teaching with both NobleStitch EL and APO, albeit APO requires major drug prescriptions. As for procedure time, we observed a long time for NobleStitch EL, which was 58 (40–75) min as compared to 30–40 min for APO. In conclusion, after the first period of technology introduction, involving the first implantations performed, no organizational impact is expected.

Both the technologies have a perceived extremely positive impact on social aspects (D8). A sensitive difference can be found in the “impact on healthcare migration,” since NobleStitch EL, performed in a few Interventional Cardiology Units in Italy according to its potentialities, represents a cause of healthcare migration, as compared to the gold standard APO, implanted in most hospitals.

Analyzing the legal aspects (D9), the two technologies obtained a similar ranking in most investigated items, as both are authorized internationally and have the Conformitè Europëenne (CE) Mark, and their user manuals are considered clear and complete by most experts. Since NobleStitch EL is currently less implanted worldwide, consulted experts believe that it should be incorporated in national and international registries to obtain further data on the device and its long-term impact. The results are shown in Table 4.

### 3.7. D10—Political and Strategic Aspects

A tertiary hospital with a significant interest in PFO treatment should consider the use of these innovative technologies, and they should contribute to further defining target populations and producing clinical efficacy and safety data, with the aim to offer a more effective, innovative, forefront, and cost-effective healthcare service.

Furthermore, healthcare professionals may benefit from the use of NobleStitch EL by attending specific training courses for its use, as their participation in stimulating training could lead to an improvement in scientific deliverables and high-level research, meeting the needs of many medical doctors and contributing to professional satisfaction. All of these factors might reflect better clinical management and patient satisfaction.

## 4. Discussion

This HTA report used original clinical and economic data, literature data derived from two systematic reviews, and expert opinion collected with semi-structured interviews to perform a multidimensional evaluation of NobleStitch EL compared to Amplatzer PFO Occluder (APO). The innovative suture-mediated NobleStitch EL device might be considered a potential turning point in symptomatic PFO treatment, allowing the closure of PFO without a device implant. Original data showed a good safety profile for NobleStitch EL (9% rhythm disturbances) as compared to APO literature data (1.9–10.1% atrial fibrillation [12,14,15,16,17,19]), as well as for device-related complications (0–5% APO [1,15,16,18] vs. 0% NobleStitch EL [11] in literature data). The efficacy profile, analyzed with the 3-month effective PFO closure, was 84% in NobleStitch EL original data (3-month TEE), 89% in literature data (3-month TTE) [11], and higher values for APO ranging from 89% to 97% (6-month TEE) [1,13,15,16,17,18,19]. Our experts are focusing on patients’ selection criteria, since they hypothesize that some morphologies of the PFO might positively affect NobleStitch EL efficacy, making it comparable to APO.

Despite the higher cost of the device and disposables than the comparator (€1.582 higher than APO) and a sensitive impact on the budget (estimated at +2.77–12.47% for PFO closure procedures, see Section 3.5 D5—Economic Financial Impact), NobleStitch EL adoption could provide benefits in terms of social and ethical aspects, without affecting organizational and legal dimensions. Our experts also underlined that a tertiary and a forefront hospital should continue to deepen this technology and provide robust data to the scientific community (see Section 3.6—Qualitative Domains D6–D9)

The limits of the present study are related to the absence of original data for APO, excluding the randomization or even a head-to-head comparison between the devices. Even the consulted literature fails to show direct comparisons. Secondly, the original data are based on a low sample of patients with a short follow-up period, so cost-effectiveness/cost-utility analysis could not be performed at this stage. Another limit is related to the difference in the sensitivity for TTE and TEE, making it difficult to compare data collected with different procedures [38]. Moreover, the answers of our professionals derive from a single academic center, thus it would be useful to integrate data with experts from other hospitals. Finally, the innovative technology, by definition, lacks long-term cost-effectiveness/benefit data that, whenever available, might significantly alter the profile of the device.

## 5. Conclusions

We suggest that there is inadequate evidence to conclude the relative efficacy of NobleStitch EL as compared to APO. Based on published early data, NobleStitch EL does not raise safety concerns and the theoretical long-term benefit of the device warrant is an ongoing evaluation. Further data are needed to directly compare these two devices and explore NobleStitch EL long-term safety/efficacy balance as well as its cost-effectiveness and cost-utility.

Research should also focus on identifying factors that may improve clinical, economic, and other relevant outcomes, and include Patient-Reported Outcomes Measures (PROMs) and Patient-Reported Experience Measure (PREMs) in a multidisciplinary evaluation [39].

## Figures and Tables

**Table 1:** Data sources used for each dimension.

Dimension	Original Clinical Data from HSR	Systematic Reviews of the Literature	Validated and ad Hoc Developed Questionnaires	Original Economic and Costs Data from HSR
D3—Safety	X	X	X	-
D4—Clinical effectiveness	X	X	X	-
D5—Economic Financial impact	-	-	-	X
D6—Ethical impact	-	-	X	-
D7—Organizational impact	-	-	X	-
D8—Social impact	-	-	X	-
D9—Legal impact	-	-	X	-

Information used for D1, D2, D10 is derived from the technical data sheet, narrative review of the literature, and expert opinion. HSR = San Raffaele Hospital.

**Table 2:** Comparison between NobleStitch EL and Amplatzer PFO Occluder.

	Amplatzer PFO Occluder	NobleStitch EL
FDA approval (year)	1998	2017
Access	Percutaneous transfemoral 8-Fr to 9-Fr	Percutaneous transfemoral 12-Fr
Kind of device	Self-expandable, double-disc device made from a platinum-filled, nickel–titanium wire mesh	No device implanted only suture stitch
Procedural time	Approximately 30–40 min	Approximately 50 min
Post-procedure antiplatelet therapy	6-month dual antiplatelet therapy	1-month single antiplatelet therapy at the discretion of the surgeon
Other characteristics	Well-known Fast Implant High efficacy	No device implanted (lower risk of allergies, arrhythmias, endocarditis, embolization, atrial or aortic wall erosion) Easy cardiac access in the future

FDA = Food and Drug Administration, PFO = Patent Foramen Ovale.

**Table 3:** Economic Analysis–Annual costs.

Process Analysis–Aggregated Costs						
	NobleStitch EL			Amplatzer PFO Occluder		
Drugs and disposable	6226.00 €			4646.53 €		
Blood components and blood products	0 €			€		
Personnel (MD, nurse, admistrative)	462.00 €			462.00 €		
Diagnostic tests	189.00 €			189.00 €		
Direct and general hospital costs	1843.04 €			1843.04 €		
Total	8720.04 €			7137.57 €		
Budget Impact Analysis						
Scenarios	Devices	Population Size	Costs	Cumulative Costs	Budget Impact (Absolute Values)	Difference (%)
As is Scenario	Amplatzer PFO	80	57,100,576 €	571,005.76 €	0.00 €	0.00%
	NobleStitch EL	0	0 €			
Innovative Scenario 1	Amplatzer PFO	70	49,963,004 €	586,830.40 €	1,582,464 €	2.77%
	NobleStitch EL	10	8,720,036 €			
Innovative Scenario 2	Amplatzer PFO	60	42,825,432 €	602,655.04 €	3,164,928 €	5.39%
	NobleStitch EL	20	17,440,072 €			
Innovative Scenario 3	Amplatzer PFO	50	35,687,860 €	61,847,968 €	4,747,392 €	7.88%
	NobleStitch EL	30	26,160,108 €			
Innovative Scenario 4	Amplatzer PFO	40	28,550,288 €	63,430,432 €	6,329,856 €	10.23%
	NobleStitch EL	40	34,880,144 €			
Innovative Scenario 5	Amplatzer PFO	30	21,412,716 €	65,012,896 €	7,912,320 €	12.47%
	NobleStitch EL	50	43,600,180 €			

MD = Medical Doctor, PFO = Patent Foramen Ovale.

**Table 4:** Results from interviews with San Raffaele Hospital experts used for D6–D9.

Domain	Item	Amplatzer PFO Occluder (Mean)	NobleStitch EL (Mean)
D3—Safety	Perceived impact on severe adverse events	1.30	1.70
	Perceived impact on moderate adverse events	1.10	1.50
	Invasiveness of the implantation procedure	−1.30	−0.90
	General safety	2.40	2.60
	Improvement in safety and tolerability	2.10	2.40
	Improvement in patient-reported outcomes	2.00	2.10
	Impact on the management of the associated drug therapy	−2.10	0.00
D6—Ethical Impact	Safeguard of patient’s autonomy	2.90	3.00
	Safeguard of human dignity and patient’s self-determination	3.00	3.00
	Safeguard of patient’s social values and willingness to pay	1.90	1.90
	Social safeguard of protected categories	3.00	3.00
	Impact on social costs	0.70	1.00
	Level of understanding of the technology by patients	2.30	2.30
	Impact on patient’s quality of life	2.20	2.70
	Impact on care-giver’s quality of life	0.80	0.90
D7—Organizational Impact—Short Term	Need for additional staff	0.40	0.50
	Need for training of the staff responsible for conducting device implant	−1.20	−1.80
	Need for training of the support staff	1.00	1.10
	Need for training of patient and care-giver	0.10	0.20
	Need for meetings, after the introduction of the technology	0.30	0.40
	Learning curve	−0.30	−0.80
	Impact on linking processes between departments	0.10	0.30
	Impact on PDT/PDTA (clinical pathways)	0.30	0.80
	Usability degree in every Interventional Cardiology Unit	0.00	0.10
D7—Organizational Impact—Long Term	Need for additional staff	0.40	0.50
	Need for training of the staff responsible for conducting device implant	0.20	0.20
	Need for training of the support staff	1.00	1.10
	Need for training of patient and care-giver	0.10	0.20
	Need for meetings, after the introduction of the technology	0.30	0.40
	Learning curve	0.00	0.00
	Impact on linking processes between departments	0.10	0.30
	Impact on PDT/PDTA (clinical pathways)	0.30	1.00
	Usability degree in every Interventional Cardiology Unit	0.00	0.10
D8—Social Aspect	Accessibility of the technology to the general population	2.50	2.20
	Accessibility of the technology to protected categories	2.30	2.30
	Impact on waiting lists	0.80	0.80
	Impact on healthcare migration	0.80	−2.50
	Existence of factors that could prevent a group from benefitting from the technology	0.00	0.10
	Impact of the patient’s willingness to pay on the accessibility of the technology	0.30	0.50
	General level of equity for the target population	2.60	2.40
D9—Legal Aspect	Authorization level	0.80	0.70
	Need for incorporation of the technology into a register	0.60	1.40
	Fulfillment of the safety requirements	2.90	2.70
	Production guarantees	2.30	2.30
	Need for price control	0.50	0.50
	Need for use regulation	0.60	0.60
	Level of legal coverage for all the user categories	1.80	2.10
	Level of thoroughness of user’s manual/IFU	2.20	2.00

## Data Availability

All the data are available in the Supplementary Material.

## Supplementary Materials

The following supporting information can be downloaded at: https://www.mdpi.com/article/10.3390/ijerph19137863/s1, Table S1—Overview of randomized clinical trials of percutaneous patent foramen ovale closure. Table S2—PFO Closure at IRCCS San Raffaele Hospital. Table S3—Authorization, regulatory status, and registration of NobleStitch EL. Table S4—Baseline echocardiographic characteristics. Table S5—PICOS First Systematic Review. Table S6—PICOS Second Systematic Review. Table S7—Characteristics of included studies, First Systematic Review NobleStitch EL. Table S8—Characteristics of included clinical trials, First Systematic Review NobleStitch El. Table S9—Procedural and in-hospital outcomes. Table S10—NobleStitch EL safety outcomes. Table S11—Definitions of outcomes used in this report. Table S12—Efficacy Outcomes. Table S13—Efficacy Outcomes Amplatzer PFO Occluder. Table S14—Follow-up transesophageal echocardiography outcomes. Table S15—Comparison of patients with and without significant residual right-to-left shunt at follow-up. Table S16—Echocardiographic predictors of ineffective PFO closure. Table S17—Echocardiographic predictors of incomplete PFO closure. Table S18—Follow-up clinical outcomes. Table S19—Bibliographic research: First Systematic Review NobleStitch EL. Table S20—Bibliographic research: Second Systematic Review Amplatzer PFO Occluder. Table S21—Characteristics of included studies, Second Systematic Review Amplatzer PFO Occluder. Table S22—Quality Appraisal for Cohort and Case-Control studies applying New Ottawa Scale. Table S23—Quality Appraisal for the Randomized Clinical Trial study applying RoB-2 scale. Table S24—Amplatzer PFO Occluder safety outcome. Table S25—Profiles of Professionals interviewed for Qualitative Domains. Figure S1—The NobleStitch EL system kit. Figure S2—The NobleStitch EL procedure. Figure S3—The NobleStitch EL procedure step by step. Figure S4—Baseline clinical characteristics. Figure S5—Patients treated with NobleStitch EL at IRCCS San Raffaele Hospital. Figure S6—Screening PRISMA of Primary literature. Figure S7—Screening PRISMA of Clinical Trials registries. Figure S8—PRISMA Flow Chart for the Second Systematic Review on Amplatzer PFO Occluder.

## Abbreviation

AGREE II = Appraisal of Guidelines for Research and Evaluation; AMSTAR 2 = Assessment of Multiple Systematic Reviews for systematic reviews and meta-analysis; APO = Amplatzer PFO Occluder; PFO = Patent Foramen Ovale; CE = Conformitè Europëenne; HSR = IRCCS San Raffaele Hospital; HTA = Health Technology Assessment; MD = Medical Doctor; PREM = Patient-Reported Experience Measure; PRISMA = Prepared Items for Systematic Reviews and Meta-Analysis; PROM = Patient-Reported Outcomes Measures; TEE = Trans-Esophageal Echocardiography; TIA = Transient Ischemic Attack; TTE = Trans-Thoracic Echocardiography; FDA = Food and Drug Administration.
