# Supplementary material for: Suture-Mediated Patent Foramen Ovale Closure Using the NobleStitch EL: Results from a Hospital-Based HTA
Source: Int J Environ Res Public Health. 2022 Jun 27;19(13):7863. doi: 10.3390/ijerph19137863 (PMC9266135; doi:10.3390/ijerph19137863)
Supplement: Supplementary file 1 [file ijerph-19-07863-s001.zip › ijerph-1718035-supplementary.pdf]

Supplementary Material

Index of Tables

Table S1 - Overview of randomized clinical trials of percutaneous patent foramen ovale closure. 3
Table S2 - PFO Closure at IRCCS San Raffaele Hospital 5
Table S3 - Authorization, regulatory status and registration of NobleStitch EL....6
Table S4 - Baseline echocardiographic characteris 12
Table S5 - PICOS First Systematic Review 12
Table S6 - PICOS Second Systematic Review....13
Table S7 - Characteristics of included studies, First Systematic Review NobleStitch EL....16
Table S8 - Characteristics of included clinical trials, First Systematic Review NobleStitch EL 16
Table S9 - Procedural and in-hospital outcomes 17
Table S10 - NobleStitch EL safety outcomes....18
Table S11 - Definitions of outcomes used in this report 18
Table S12 - Efficacy Outcomes....19
Table S13 - Efficacy Outcomes Amplatzer PFO Occluder 20
Table S14 - Follow-up transoesophageal echocardiography outcomes....21
Table S15 - Comparison of patients with and without significant residual right-to-left shunt at follow-up. 22
Table S16 - Echocardiographic predictors of ineffective PFO closure 23
Table S17 - Echocardiographic predictors of incomplete PFO closure....23
Table S18 - Follow-up clinical outcomes....24
Table S19 - Bibliographic research: First Systematic Review NobleStitch EL 25
Table S20 - Bibliographic research: Second Systematic Review Amplatzer PFO Occluder 25
Table S21 - Characteristics of included studies, Second Systematic Review Amplatzer PFO Occluder....26
Table S22 - Quality Appraisal for Cohort and Case-Control studies applying New-Ottawa Scale 27
Table S23 - Quality Appraisal for the Randomized Clinical Trial study applying RoB-2 scale....27
Table S24 - Amplatzer PFO Occluder safety outcome 28
Table S25 - Profiles of Professionals interviewed for Qualitative Domains....30

Index of Figures

Figure S1 - The NobleStitch EL system kit 7
Figure S2 - The NobleStitch EL procedure 7
Figure S3 - The NobleStitch EL procedure step by step 8
Figure S4 - Baseline clinical characteristics. 10
Figure S5 - Patients treated with NobleStitch EL at IRCCS San Raffaele Hospital 11
Figure S6 - Screening PRISMA of Primary literature. 14
Figure S7 - Screening PRISMA of Clinical Trials registries 14
Figure S8 - PRISMA Flow Chart for the Second Ststematic Review on Amplatzer PFO Occluder 15

Table S1 - Overview of randomized clinical trials of percutaneous patent foramen ovale closure.

Study	Design	Inclusion criteria	Endpoints	F/U	Results	Comments
CLOSURE-I (2012)	STARFlex Septal Closure System plus DAPT vs. ASA and/or VKA<br><br>Superiority RCT 1:1<br><br>N=909<br>PFO closure=447<br>Medical Tx=462<br><br>Enrolled: 2003-2008	Age: 18-60 years with cryptogenic stroke/TIA/SE within 6 months<br>*and*<br>TOE verified PFO<br><br>Moderate/large shunt*: 55.9%<br>Atrial septal aneurysm†: 37.6%	Primary endpoint: stroke/TIA/early death/late neurologic death<br><br>Grade of residual shunt:<br>0: no bubbles;<br>1: 1-10 bubbles;<br>2: 11-25 bubbles;<br>3: >25 bubbles within 5 cardiac cycles	2	Primary endpoint: 5.5% vs. 6.8% (HR 0.78, 95% CI 0.45-1.35, *p*=0.37)<br>Stroke: 2.9% vs. 3.1% , *p*=0.79<br>TIA: 3.1% vs. 4.1%, *p*=0.44<br><br>Procedural success: 89.4%<br>Effective closure at 6-month TOE (grade ≤1 residual shunt): 86.1%<br>Major bleeding: 2.6% vs. 1.1%, *p*=0.11<br>Major vascular complications: 3.2% vs. 0%, *p*<0.001<br><br>AF: 5.7% vs. 0.7%, *p*<0.001<br>Device thrombosis: 1.1%, 6-month TOE (50% associated w recurrent stroke)	*Device closure*: Clopidogrel 75 mg plus ASA 81-325 mg for 6 months, then ASA<br>*Medical therapy*: ASA 325 mg and/or VKA (INR 2.0-3.0)<br><br>No interaction between atrial septal aneurysm or baseline shunt and primary endpoint¶
PC Trial (2013)	Amplatzer PFO Occluder plus DAPT vs. ASA and/or VKA<br><br>Superiority RCT 1:1<br><br>N=414<br>PFO closure=204<br>Medical Tx=210<br><br>Enrolled: 2000-2009	Age: <60 years with cryptogenic stroke/TIA/SE<br>*and*<br>TOE verified PFO<br><br>Large shunt*: 23.2%<br>Atrial septal aneurysm†: 23%	Primary endpoint: death/non-fatal stroke/TIA/SE<br><br>Grade of residual shunt:<br>0: no bubbles<br>1: 1-5 bubbles;<br>2: 6-20 bubbles<br>3: >20 bubbles	4.1	Primary endpoint: 3.4% vs. 5.2% (HR 0.63, 95% CI 0.24-1.72, *p*=0.34)<br>Stroke: 0.5% vs. 2.4%, *p*=0.14<br>TIA: 2.5% vs. 3.3%, *p*=0.56<br><br>Procedural success 95.9%<br>Effective closure at 6-month TOE (grade ≤1 residual shunt): 95.9% (142/148)<br>Major bleeding: 0.5% vs. 1.4%, *p*=0.62<br><br>AF: 2.9% vs 1.0%, *p*=0.16<br>Device thrombosis: 0%	*Device closure*: Clopidogrel 75-150 mg or Ticlopidine 250-500 mg plus ASA 100-325 mg for 1-6 months<br>*Medical therapy*: ASA 325 mg and/or VKA (INR 2.0-3.0)<br><br>Trend toward interaction between atrial septal aneurysm and primary endpoint¶
RESPECT (2013)	Amplatzer PFO Occluder plus DAPT vs. APT or VKA<br><br>Superiority RCT 1:1	Age 18-60 years with cryptogenic stroke/TIA/SE within 9 months<br>*and*<br>TOE verified PFO<br>Large shunt*:	Primary endpoint: fatal or non-fatal ischemic stroke/early death	2.6	Primary endpoint: 1.8% vs. 3.3% (HR 0.49, 95% CI 0.22-1.11, *p*=0.08)<br>All events were non-fatal strokes<br><br>Procedural success 96.1%<br>Complete closure at 6-month TOE	*Device closure*: Clopidogrel 75 mg plus ASA 81-325 mg for 1 month, then ASA for 5 months<br>*Medical therapy*:

	N=980<br>PFO closure=499<br>Medical Tx=481<br><br>Enrolled: 2003-2011	49.5%<br>Atrial septal aneurysm†: 36.1%	Grade of residual shunt:<br>0: no bubbles<br>1: 1-9 bubbles;<br>2: 10-20 bubbles<br>3: >20 bubbles<br>within 3 cardiac cycles		(grade 0 residual shunt): 72.7%<br>Effective closure at 6-month TOE (grade ≤1 residual shunt): 93.5%<br>Major bleeding 0.4%<br>Major vascular complications: 0.6%<br><br>TIA: 1.2% vs 0.8%, *p*=0.83<br>PE: 1.2% vs. 0.2%, *p*=0.12<br>AF: 3.0% vs. 1.5%, *p*=0.13<br>Device thrombosis: 0.2%	ASA or Clopidogrel or ASA plus Dipyridamole or VKA (INR 2.0-3.0)<br><br>Device superiority in the pre-specified per-protocol and as-treated analysis
CLOSE (2017)	PFO closure using different devices (Amplatzer PFO Occluder 51.5%) plus DAPT vs. APT<br>*and*<br>APT vs. OAC<br>Superiority RCT 1:1:1<br>Investigator-initiated<br><br>N=660<br>PFO Closure=238<br>Medical Tx=422<br>Enrolled: 2008-2016	Age 16-60 years with cryptogenic stroke within 6 months<br>*and*<br>TOE verified high-risk PFO (atrial septal aneurysm or large shunt)<br><br>Large shunt*: 91%<br>Atrial septal aneurysm†: 34%<br><br>RoPE score 7.4±1.3	Primary endpoint: fatal or non-fatal stroke<br><br>Grade of residual shunt:<br>0: no bubbles<br>1: 1-10 bubbles;<br>2: 11-30 bubbles<br>3: >30 bubbles<br>within 3-5 cardiac cycles	5.3	Primary endpoint: 0% vs. 4.9% (HR 0.03, 95% CI 0-0.26, *p*<0.001)‡<br><br>NNT=20<br><br>Procedural success 99.6%<br>Effective closure at 12-month TOE (grade ≤1 residual shunt): 93% (212/228)<br>Major bleeding: 0.8% vs 2.1%, *p*=0.28<br><br>TIA: 3.3% vs. 3.4%, *p*=0.96<br>AF: 4.6% vs 0.9%, *p*=0.02<br>Device thrombosis: 0.4%	*Device closure*: Clopidogrel 75 mg plus ASA 75 mg for 3 months, then ASA or Clopidogrel or ASA plus Dipyridamole<br>*APT*: ASA or Clopidogrel or ASA plus Dipyridamole<br>*OAC*: VKA (INR 2.0-3.0) or NOACs<br>Trend toward interaction between atrial septal aneurysm or baseline large shunt and primary endpoint¶
Gore REDUCE (2017)	Helex Septal Occluder (HELEX) or Cardioform Septal Occluder (GSO) plus APT vs. APT<br><br>Superiority RCT 2:1<br><br>N=664<br>PFO Closure=441<br>Medical Tx=223<br><br>Enrolled: 2008-2015	Age: 18-59 years with cryptogenic stroke within 6 months<br>*and*<br>TOE verified PFO<br><br>Large shunt*: 42.8%<br>Atrial septal aneurysm†: 20.4%	Primary endpoint: clinical ischemic stroke<br>Co-primary endpoint: clinical ischemic stroke or silent brain infarction<br><br>Grade of residual shunt:<br>0: no bubbles;<br>1: 1-5 bubbles;<br>2: 6-25 bubbles;<br>3: >25 bubbles<br>within 3 cardiac cycles	3.2	Primary endpoint: 1.4% vs 5.4% (HR 0.23, 95% CI 0.09-0.62, *p*=0.002)<br>Co-primary endpoint: 5.7 vs. 11.3 (HR 0.51, 95% CI 0.29-0.91, *p*=0.04)<br>NNT=28<br>Procedural success 98.8%<br>Complete closure at 12-month TOE (grade 0 residual shunt): 75.6%<br>Effective closure at 12-month TOE (grade <3 residual shunt): 94.5%<br>Major bleeding: 0.9%<br>TIA: 0.2% vs. 0.4%, *p*=1<br>AF: 6.6% vs. 0.4%, *p*<0.001<br>Device thrombosis: 0.5%	*Both groups*: Clopidogrel 75 mg or ASA 75-325 mg or ASA 50-100 mg plus Dipyridamole 225-400 mg<br><br>Trend toward interaction between moderate to large baseline shunt and primary endpoint

RESPECT Long Term<br>(2017)	Amplatzer PFO Occluder plus DAPT<br>vs.<br>APT or VKA<br>Superiority RCT 1:1<br>N=980<br>- PFO Closure=499<br>- Medical Tx=481<br><br>Enrolled: 2003-2011<br>Ext. F/U to 2016	Age 18-60 years with cryptogenic stroke within 9 months<br>*and*<br>TOE verified PFO<br><br><br>Large shunt*:<br>49.5%<br>Atrial septal aneurysm†:<br>36.1%	Primary endpoint:<br>fatal/non-fatal stroke/early death<br><br>Grade of residual shunt:<br>0: no bubbles<br>1: 1-9 bubbles;<br>2: 10-20 bubbles<br>3: >20 bubbles<br>within 3 cardiac cycles	5.9	Primary endpoint: 3.6% vs. 5.8%<br>0.58 vs. 1.07 events/100 patient-yr<br>(HR 0.55, 95% CI 0.31-0.99, p<0.046)<br><br>NNT=42<br><br>TIA: 0.54 vs. 0.86 /100 patient-yr, p=0.16<br>PE: 0.41 vs. 0.11 /100 patient-yr, P=0.04<br>AF: 0.48 vs. 0.34 /100 patient-yr, P=0.36<br>Device thrombosis: 0.4%	*Device closure*:<br>Clopidogrel 75 mg plus ASA 75 mg for 1 month, then ASA for 5 months<br>*Medical therapy*:<br>ASA or Clopidogrel or ASA plus Dipyridamole or VKA (INR 2.0-3.0)
DEFENSE PFO<br>(2018)	Amplatzer PFO Occluder plus APT or VKA vs.<br>APT or VKA<br>Superiority RCT 1:1<br>Investigator-initiated<br>N=120<br>- PFO Closure=60<br>- Medical Tx=60<br><br>Enrolled: 2011-2017	Age 18-80 years with cryptogenic stroke within 6 months *and*<br>TOE verified high-risk PFO (large or hypermobile PFO or atrial septal aneurysm)<br><br>Large shunt*: 58.3%<br>Atrial septal aneurysm†:<br>8.3%	Primary endpoint:<br>stroke/vascular death/major bleeding	2.8	Primary endpoint: 0% vs. 12.9%<br>(95% CI 3.2-22.6, p=0.013)<br>Stroke: 0% vs 10.5%<br>(95% CI 1.6-19.3, p=0.023)<br>Major bleeding: 0% vs 4.9%, p=0.15<br><br>NNT=10 (for stroke prevention)<br><br>Procedural success 100%<br>Effective closure at 24-hours TTE (grade ≤1 residual shunt): 93.3% (56/60)<br><br>Silent brain infarction at 6-month MRI:<br>8.8% vs.18.4%, p=0.24<br>TIA 0% vs. 2%, p=0.32<br>AF: 3%<br>Device thrombosis: 0%	*Device closure*:<br>Clopidogrel 75 mg plus ASA 100 mg for 6 months (recommended)<br>*Both groups*:<br>ASA 100 mg, or<br>ASA 100 mg plus Clopidogrel 75 mg, or<br>ASA 100 mg plus Cilostazol 200 mg, or<br>VKA (INR 2.0-3.0)<br><br>Early trial termination for patient safety, resulting in underpower and inability to provide HR

ASA=acetylsalicylic acid; DAPT=dual antiplatelet therapy; NOAC=novel oral anticoagulant; PFO=patent foramen ovale.

Table S2 - PFO Closure at IRCCS San Raffaele Hospital

	**Jul-2017 – Jun-2018 (12 months)**	**Jul-2018 – Aug-2019 (14 months)**
**Total N patients undergone PFO Closure**	77	86
**N patients undergone PFO Closure with NobleStitch EL**	29 out of 77	36 out of 86
**% of NobleStitch EL**	38%	42%
**% of Amplatzer PFO Occluder**	62%	68%

Table S3 - Authorization, regulatory status and registration of NobleStitch EL

**Device type**	Device
**Registry Identification Database (Banca dati - BD)/Medical Devices Inventory (Repertorio Dispositivi Medici - RDM)**	1398635
**Inventory**	S
**Code attributed by the producer**	12-90-X7X
**Commercial name and model**	NobleStitch EL
**National Classification of Medical Devices (Classificazione Nazionale dei Dispositivi medici - CND)**	p07040303
**CE Class**	III
**First Publication Date**	31/03/2016
**Firm role**	Producer
**Denomination**	Nobles Medical Technologies Ii, Inc.
**Vat number**	-
**Country**	US

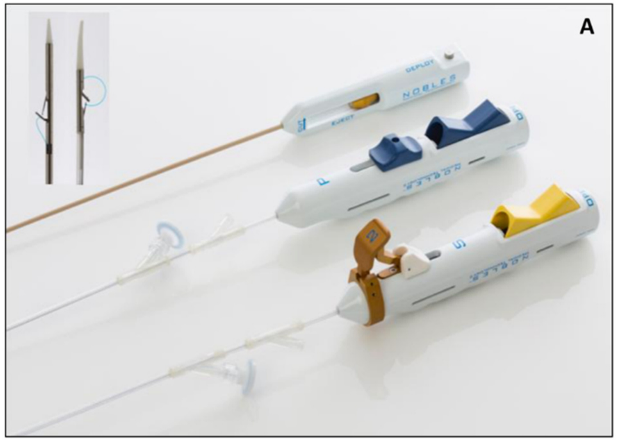

Figure S1 - The NobleStitch EL system kit ©

Image available at https://www.kardia.it/dispositivo_medico/noblestitch-el/

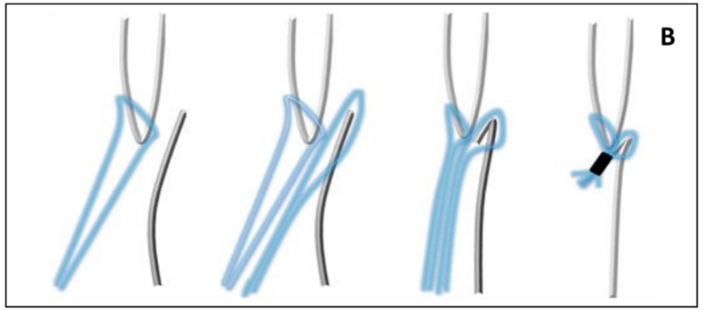

Figure S2 - The NobleStitch EL procedure

Image available at https://www.kardia.it/dispositivo_medico/noblestitch-el/

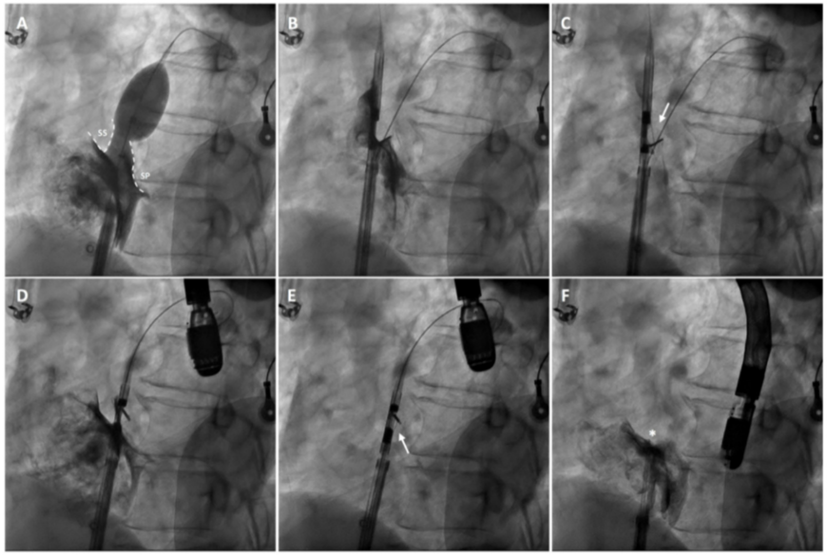

Figure S3 - The NobleStitch EL procedure step by step

Detailed procedure: *The procedure is performed under local anesthesia and fluoroscopic guidance, with or without TOE monitoring. When TOE monitoring is required, patients might receive general anesthesia with propofol, orotracheal intubation and mechanical ventilation for the duration of the procedure. The right femoral vein is used as the preferential vascular access site for the procedure. A multipurpose or similar catheter is advanced to cross the PFO and a 0.035'' stiff guidewire is placed in the left upper pulmonary vein. After exchanging the multipurpose catheter for a straightened 14 Fr Mullins sheath, sizing balloon interrogation of the PFO during contrast injection is performed to determine the anatomy of the septum secundum and septum primum (**Figure 3A**). Subsequently, the 0.035'' stiff guidewire is exchanged for a 0.032'' guidewire and an additional 0.018" guidewire is placed into the distal superior vena cava. The NobleStitch S and P are then sequentially advanced to suture the septum secundum (**Figure 3B-C)** and the septum primum **(Figure 3D-E**), respectively, with special care taken to puncture the septum primum at the nadir of the septum secundum. Contrast injections are carried out according to operator discretion to obtain optimal engagement of each septum. After each NobleStitch needle has been advanced through the septum to capture and retrieve the suture, the system is removed along with the wire, leaving the sutures free. Then, the suture ends are gently pulled together to remodel the septum primum, drawing it towards the right atrium and closing the PFO. Maintaining the tension on the sutures, the KwiKnot delivery catheter is then used to advance and release a radiopaque polypropylene knot on the right side of the atrial septum and to cut the proximal suture. Contrast injection and/or TOE or TTE saline microbubbles test are performed in all patients to assess the acute result **(Figure 3F).** In case of device failure, the use of a second stitch or a bailout traditional PFO closure device might be permitted, according to operator's choice, device availability, and patient's specific atrial septum anatomy. Prophylactic antibiotics (single pre-procedural dose of intravenous cefazolin*

2 g or clindamycin 600 mg) are administered in all patients. Patients are pre-treated with a single antiplatelet agent (aspirin 100 mg daily or clopidogrel 75 mg daily) in the absence of other indications, and receive a minimum 100 IU/kg of unfractionated heparin at the beginning of the procedure, followed by further boluses in order to maintain an activated clotting time constantly >250 seconds. During in-hospital stay patients received 24-hour ECG monitoring after the procedure. Renal function is not routinely assessed after the procedure, unless clinically necessary. Early patient mobilization and discharge are encouraged. At discharge, antiplatelet therapy continuation is left to the discretion of the attending physician. In the absence of other indications, the standard protocol consists in a single antiplatelet agent (aspirin 100 mg) daily for one month. Antibiotic prophylaxis is advised for five days after discharge.

Clinical Characteristics	Patients (N=65)
Age (years)	48±12
Female	35 (54)
Medical history	
Hypertension	17 (26)
Dyslipidemia	20 (31)
Current smoker	9 (14)
Diabetes	2 (3)

Reason for PFO closure<br>Recurrent cryptogenic cerebrovascular accident<br>Intractable migraine<br>Professional scuba diving<br>Intracranial surgery requiring sitting anesthesia/ventilation	 55 (84)<br>8 (12)<br>1 (1.5)<br>1 (1.5)
Type of recurrent cryptogenic cerebrovascular accident<br>Ischemic stroke<br>TIA	 29 (53)<br>26 (47)
Disabling stroke (modified Rankin scale* ≥3 at 6 months)	0 (0)
RoPE score†	5 (4-7)

Figure S4 - Baseline clinical characteristics.

Data are presented as absolute numbers and percentages (for categorical variables) or median value (IQR) or mean value±SD (for continuous variables), as appropriate.

*The modified Rankin scale is a measure of disability. Scores range from 0 (no symptoms) to 6 (death). A score of 3 or higher indicates at least moderate disability, with the need for some help in daily affairs.

†Risk of Paradoxical Embolism (RoPE) is a score index used to indicate whether a PFO in cryptogenic cerebrovascular accidents is stroke/TIA-related or incidental. Scores range from 0 to 10, with larger values representing a higher probability that a PFO is related to cryptogenic cerebrovascular accident.

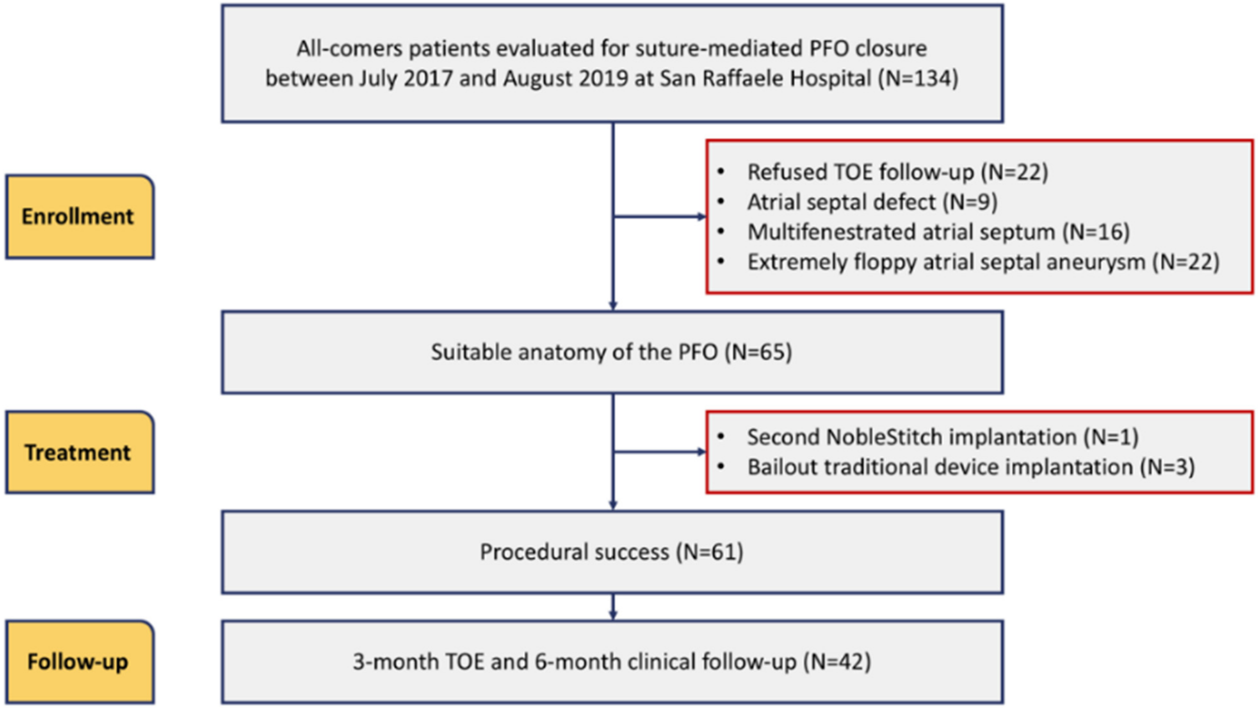

Figure S5 - Patients treated with NobleStitch EL at IRCCS San Raffaele Hospital

Table S4 - Baseline echocardiographic characteristics

Echocardiographic Characteristics	Patients (N=65)
PFO length (mm)	10 (8-12)
PFO diameter (mm)	3 (2-4)
Atrial septal aneurysm*	6 (10)
Prominent eustachian valve	8 (12)
Baseline color shunt (stretched PFO)†	6 (10)
Valsalva color shunt	7 (11)
Baseline positive microbubbles test	33 (51)
Valsalva positive microbubbles test	65 (100)
Right-to-left shunt severity<br>Grade 2 shunt<br>Grade 3 shunt	 31 (48)<br>34 (52)
High-risk PFO‡	46 (70)

Data are presented as absolute numbers and percentages (for categorical variables) or median value (IQR) (for continuous variables), as appropriate.

* Atrial septal aneurysm was defined as excursion of the septal tissue of >10 mm from the plane of the atrial septum into the right or left atrium or a combined total excursion right and left of ≥15 mm.

†Stretched PFO derives from stretching of the superior limbic band of the septum secundum by atrial dilation and remodelling, without a true deficiency of the atrial septal tissue, resulting in baseline color-Doppler left-to-right shunt.

‡High-risk PFO is defined as the presence of atrial septal aneurysm and/or moderate to severe shunt (grade ≥2) and refers to the risk of paradoxical embolization.

Table S5 - PICOS First Systematic Review

**Population**	Patients suffering from symptoms related to PFO
**Intervention**	NobleStitch EL
**Comparison/control**	Amplatzer PFO Occluder literature data, no direct comparison
**Outcomes**	Any relevant outcome

**Studies**	Retrospective and prospective observational studies (cohort and case-control studies), randomized controlled trials (RCTs), non-randomized controlled trials, economic studies, systematic reviews, meta-analysis, guidelines, health technology assessment reports.

Table S6 - PICOS Second Systematic Review

**Population**	Patients suffering from symptoms related to PFO
**Intervention / Comparator**	Amplatzer PFO Occluder
**Outcomes**	Efficacy: Effective PFO Closure<br>Safety: Any adverse event
**Studies**	Retrospective and prospective observational studies (cohort and case-control studies), randomized controlled trials (RCTs), non-randomized controlled trials, economic studies. – with at least 50 patients.

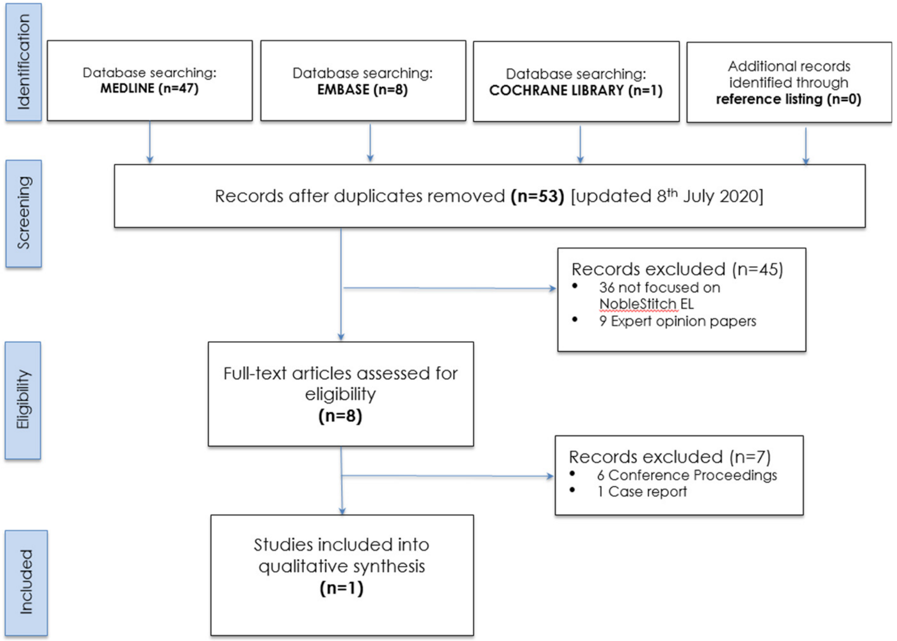

Figure S6 - Screening PRISMA of Primary literature.

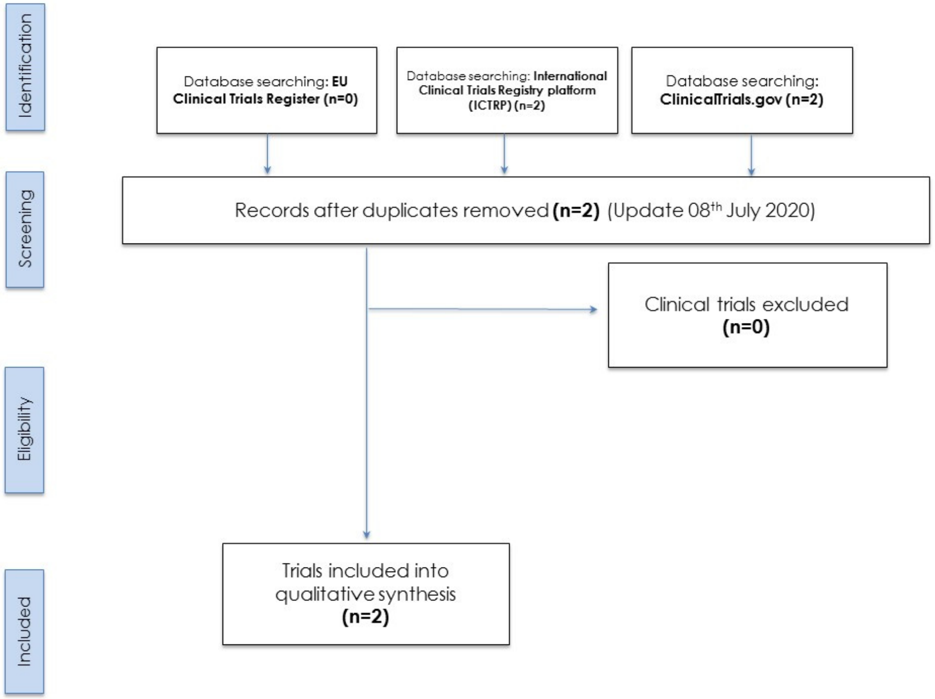

Figure S7 - Screening PRISMA of Clinical Trials registries

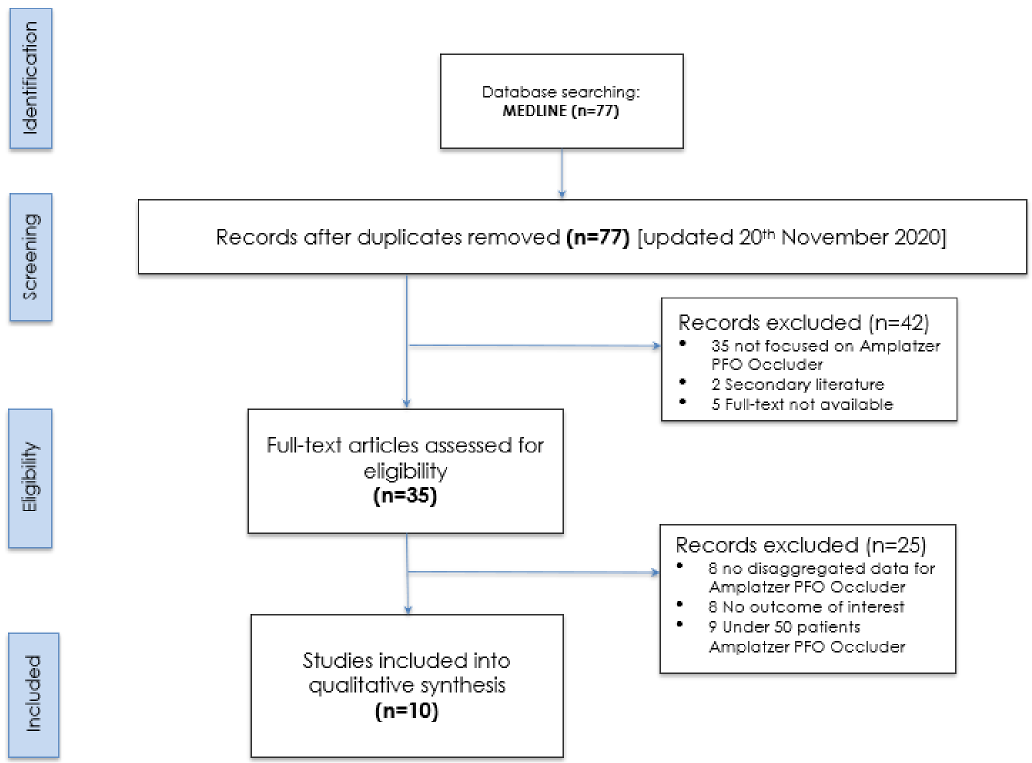

Figure S8 - PRISMA Flow Chart for the Second Ststematic Review on Amplatzer PFO Occluder

Table S7 - Characteristics of included studies, First Systematic Review NobleStitch EL

N°	Ref (first author)	Year	Journal	Country	Study design	Sample size	Intervention	Comparator	Follow-up
**1**	Gaspardone A**.** (1)	2018	*EuroIntervention*	Italy, UK, Germany	Single-arm observational study (Journal Article)	200	NobleStitch EL	No comparator	206±130 days

Table S8 - Characteristics of included clinical trials, First Systematic Review NobleStitch EL

	Protocol number	Primary sponsor	Country of implementation	Date of autorization/ Registration	Date of 1st enrolement	Sample size	Clinical conditions	Population age	Intervention	Comparator	Primary outcomes
**1**	NCT04339699	HeartStitch. Com	USA and the European Union	April 9, 2020	Not yet recruiting Estimated Date: June 1, 2020	640 participants	PFO Stroke, Ischemic	18 - 60 years	NobleStitch EL Suture	FDA Approved Amplatzer Occluder Device	Effective PFO closure rate of the NobleStitch EL [6 months]
**2**	NCT03373929	HeartStitch. Com	Inova Cardiovascular Institute Falls Church, Virginia, United States	December 14, 2017	Start Date: November 20, 2017	250 participants	PFO, Septal Defect, Atrial Septal Defect, Heart	18 - 65 years	Device: PFO Closure Rate Device: Published PFO Device Closure	\	Closure Rates of PFO and ASD [Time Frame: 12 months] Saline Contrast Echo.

Procedural and In-Hospital Outcomes	Patients (N=65)
Procedural time (min)	45 (30-64)
Radiation exposure (Gy·cm^2)	62 (40-130)
Contrast media volume (mL)	140 (100-175)
Transoesophageal echocardiography guidance	3 (5)
Successful PFO closure*	61 (94)
Second stitch delivery	1 (1.5)
Bailout traditional device†	3 (5)
Procedural complications<br>Device detachment<br>Access site minor bleeding‡<br>Retroperitoneal hemorrhage<br>Arteriovenous fistula<br>Cardiac tamponade<br>Pericardial effusion<br>Atrial fibrillation or supraventricular tachycardia	 0 (0)<br>2 (3)<br>1 (1.5)<br>1 (1.5)<br>0 (0)<br>0 (0)<br>2 (3)
Hospitalization length (days)	2.9±0.9
In-hospital all-cause death	0 (0)
Antithrombotic therapy at discharge<br>ASA<br>Clopidogrel<br>DAPT<br>NOAC<br>None	 31 (47)<br>9 (14)<br>20 (31)<br>2 (3)<br>3 (5)

Table S9 - Procedural and in-hospital outcomes in patients treated in San Raffaele Hospital

Data are presented as absolute numbers and percentages (for categorical variables) or median value (IQR) or mean value±SD (for continuous variables), as appropriate.
*Successful PFO closure was defined as correct delivery of the septal sutures followed by negative intraprocedural contrast injection and/or TOE or TTE microbubbles test.
†Amplatzer PFO Occluder (Abbott, Santa Clara, CA, USA) was used as bailout device in all the 3 cases.
‡Bleeding severity was defined according to BARC classification.
ASA=acetylsalicylic acid; DAPT=dual antiplatelet therapy; NOAC=novel oral anticoagulant; PFO=patent foramen ovale.

Table S10 - NobleStitch EL safety outcomes from the first systematic review

Safety NobleStitch EL								
**First author**	**Year**	**Study design**	**Device**	**N. pts. Inter**	**Comparator**	**Outcome**	**Results NobleStitch**	**Follow-up**
Gaspardone (1)	2018	Single-arm observational study (Journal Article)	NobleStitch EL	186	\	Procedure time	58 min (40-75)	206±130
						Contrast volume	200 ml (150-270)	206±130
						Radiation dose absorbed	87 Gy*cm2 (52-125)	206±130
						Device deployement	186/192 (89%)	206±130
						Intraprocedural complications	0/192 (0%)	206±130
						Atrial fibrillation	0/192 (0%)	206±130
						Device-related complications	0/186 (0%)**	206±130

** All complication of endovascular treatment leading to death. Cardiac perforation/tamponade requiring emergency drainage. Cerebral air embolism responsible for acute neurological disorders. Complications related to the vascular access: haematoma at the puncture site (>60 mm in diameter) or any haematoma requiring transfusion or surgical correction with or without fatal or life-threatening haemorrhage; AV fistula, pseudoaneurysm requiring surgical correction; peripheral nerve lesion with disabling neurological deficit persisting for more than one month. Any complications related to general anaesthesia or TOE. Bacterial endocarditis (fever, septicaemia, vegetations). Asymptomatic early or late intracardiac thrombosis detected periprocedurally on follow-up echocardiography. Transient or persistent arrhythmias, observed during catheterisation or post procedure, requiring or not requiring prolonged treatment. Increase in creatinine levels >20% of the basal level 24 hours after the endovascular procedure.

Table S11 - Definitions of outcomes used in this report

		**Definition**		
Microbubble Test	*RLS grade*	*Significant RLS*	*Effective PFO Closure*	*Complete PFO Closure*
Negative	0		x	x
Mild positive	1			
Moderate positive	2	x		
Severe positive	3			

Table S12 - Efficacy Outcomes from the first systematic review

EFFICACY OUTCOMES – NobleStitch EL												
**Outcome**	**First author**	**Year**	**Study Design**	**Device**	**N. pts. Inter**	**Comparator**	**N. pts. Comp**	**Results NobleStitch**	**Results on Comparator**	**US used**	**Follow-up (days)**	**p-value**
Microbubble Test Negative after follow up	Gaspardone (1)	2018	Single-arm observational study (Journal Article)	NobleStitch EL	186	\	\	139/186 (75%)	\	TTE	206±130	\
RLS ≤ 1 after follow-up	Gaspardone (1)	2018	Single-arm observational study (Journal Article)	NobleStitch EL	186	\	\	166/186 (89%)	\	TTE	206±130	\
RLS = 2 after follow-up	Gaspardone (1)	2018	Single-arm observational study (Journal Article)	NobleStitch EL	186	\	\	11/186 (5.9%)	\	TTE	206±130	\
RLS =3 at follow-up	Gaspardone (1)	2018	Journal Article	NobleStitch EL	186	\	\	9/186 (4.8%)	\	TTE	206±130	\
Significant RLS after follow up	Gaspardone (1)	2018	Single-arm observational study (Journal Article)	NobleStitch EL	186	\	\	20/186 (10.7%)	\	TTE	206±130	\

Table S13 - Efficacy Outcomes Amplatzer PFO Occluder from the second systematic review

EFFICACY OUTCOMES – Amplatzer PFO Occluder												
**Ref (First Author)**	**Year**	**Journal**	**Country Of Implementation**	**Study Design**	**Sample Size (Tot)**	**Intervention**	**N Pts Intervention**	**Control**	**N Pts Control**	**Result On Amplatzer**	**Follow-Up**	**Ecography Used**
**RLS <=1 after Follow-up**												
Scalise (2)	2016	Journal of Interventional Cardiology	Italy	Case Control	205	Amplatzer PFO Occluder	52	Figulla Flex	49	91.40%	53 months	TTE
Wahl (3)	2009	JACC: Cardiovascular Interventions	Switzerland	Cohort Study	620	Amplatzer PFO Occluder	620	/	/	97%	6 months	TEE
Luani (4)	2015	EuroIntervention	Germany	Cohort Study	335	Amplatzer PFO Occluder	109	BIOstar, Cardia, Premere	226	89%	3 months	TEE
Meier (5)	2013	The New England journal of medicine	Europe, Canada, Brazil, Australia	multicenter randomized clinical trial	414	Amplatzer PFO Occluder	204	medical therapy	210	95.9%	4.1 years	TEE
Braun (6)	2003	European Hearth Journal	Germany	Observational study	307	Amplatzer PFO Occluder	69	PFO Star occluder, CardioSeal	238	89%	21 months	TEE
Hammerstingl (7)	2011	European Journal Of Medical Research	Germany	Case Control	124	Amplatzer PFO Occluder	52	CardioSeal, Premere, Helex	72	88,50%	6 months	TEE
Trabattoni (8)	2017	EuroIntervention	Italy	Case Control	406	Amplatzer PFO Occluder	179	Figulla	227	95.50%	6 months	TTE
**RLS = 2**												
Wahl (3)	2009	JACC: Cardiovascular Interventions	Switzerland	Cohort Study	620	Amplatzer PFO Occluder	620	/	/	2%	6-month	TEE

Trabattoni (8)	2017	EuroIntervention	Italy	Case Control	406	Amplatzer PFO Occluder	179	Figulla	227	4,50%	6 months	TTE
**RLS = 3**												
Wahl (3)	2009	JACC: Cardiovascular Interventions	Switzerland	Cohort Study	620	Amplatzer PFO Occluder	620	/	/	1%	6-month	TEE
Trabattoni (8)	2017	EuroIntervention	Italy	Case Control	406	Amplatzer PFO Occluder	179	Figulla	227	0,00%	6 months	TTE

Table S14 - Follow-up transoesophageal echocardiography outcomes in patients treated in San Raffaele Hospital

Transoesophageal Echocardiography Outcomes	Patients (N=42)
Follow-up time (days)	101±38
Device detachment	0 (0)
Device thrombosis	1 (2)
Atrial septal tear*	1 (2)
Baseline color shunt	3 (7)
Valsalva color shunt	3 (7)
Baseline positive microbubbles test	3 (7)
Valsalva positive microbubbles test	12 (28)
Right-to-left shunt severity<br>Grade 0 shunt<br>Grade 1 shunt<br>Grade 2 shunt<br>Grade 3 shunt	 30 (72)<br>5 (12)<br>6 (14)<br>1 (2)
Complete PFO closure†	30 (72)
Effective PFO closure‡	35 (83)

Of the 65 patients included in the study, 42 (65%) underwent 3-month TOE follow-up.
Data are presented as absolute numbers and percentages (for categorical variables) or mean value±SD (for continuous variables), as appropriate.
*Diamond-shaped atrial septal defect with left-to-right shunt.
†Complete closure was defined as a residual right-to-left shunt grade 0 at follow-up.
‡Effective closure was defined as a residual right-to-left shunt grade ≤1 at follow-up.

Table S15 - Comparison of patients with and without significant residual right-to-left shunt at follow-up in patients treated in San Raffaele Hospital

	Effective closure (N=35)	Ineffective closure (N=7)	*p*-value
Age (years)	48±12	52±10	0.517
Female	15 (42.9)	3 (42.9)	0.656
Reason for PFO closure<br>Recurrent cryptogenic ischemic stroke<br>Recurrent cryptogenic TIA<br>Intractable migraine	 13 (37.1)<br>14 (40)<br>6 (17)	 3 (42.9)<br>4 (57)<br>0 (0)	 0.546<br>0.355<br>0.309
RoPE score*	6 (3-9)	5 (4-8)	0.604
PFO length (mm)	11 (6-22)	9.5 (6-15)	0.378
PFO diameter (mm)	3 (1-6)	3.5 (2-7)	0.961
Atrial septal aneurysm	4 (11.8)	1 (16.7)	0.577
Prominent eustachian valve	4 (11.8)	2 (33.3)	0.215
Baseline color shunt (stretched PFO)	3 (8.6)	0 (0)	0.570
Valsalva color shunt	3 (8.6)	0 (0)	0.570
Baseline microbubbles test positivity	15 (42.9)	6 (85.7)	**0.047**
Right-to-left shunt severity<br>Grade 2 shunt<br>Grade 3 shunt	 17 (48.6)<br>7 (20)	 4 (57)<br>1 (14.3)	 0.904<br>0.904
Transoesophageal echocardiography guidance	2 (5.9)	0 (0)	0.684
Procedural time (min)	50 (34-67)	33 (24-60)	0.071
Radiation exposure (Gy·cm^2)	66 (20.6-286)	104.5 (32-248)	0.246
Contrast media volume (mL)	140 (70-440)	135 (68-160)	0.261

Baseline and procedural characteristics of the patients treated with the NobleStitch EL device with and without significant (grade ≥2) residual right-to-left shunt at 3-month TOE follow-up are reported. Of the 65 patients included in the study, 42 (65%) underwent 3-month TOE follow-up.

Data are presented as absolute numbers and percentages (for categorical variables) or median value (IQR) or mean value±SD (for continuous variables), as appropriate.

Echocardiographic and clinical follow-up length were not significantly different between the two groups

*Risk of Paradoxical Embolism (RoPE) is a score index used to indicate whether a PFO in cryptogenic cerebrovascular accidents is stroke/TIA-related or incidental. Scores range from 0 to 10, with larger values representing a higher probability that a PFO is related to cryptogenic cerebrovascular accident.

Table S16 - Echocardiographic predictors of ineffective PFO closure in patients treated in San Raffaele Hospital

Variable	OR (95% CI)	*p*-value	C-statistics
PFO length	0.86 (0.63-1.18)	0.355	-
PFO diameter	1.18 (0.60-2.31)	0.632	-
Atrial septal aneurysm	1.50 (0.14-16.32)	0.739	-
Prominent eustachian valve	3.75 (0.51-27.49)	0.194	-
Baseline color shunt (stretched PFO)	4.33 (0.23-79.58)	0.323	-
Valsalva color shunt	4.33 (0.23-79.58)	0.323	-
Baseline positive microbubbles test*	8.0 (0.87-73.68)	0.066	-
Right-to-left shunt grade 2	1.27 (0.10-16.81)	0.855	-
Right-to-left shunt grade 3	1.64 (0.15-17.47)	0.679	-

Univariate binary logistic regression analysis for predictors of incomplete PFO closure (residual right-to-left shunt ≥ grade 2 at 3-month TOE follow-up) with the NobleStitch EL device.
*Positive microbubbles test during normal respiration.

Table S17 - Echocardiographic predictors of incomplete PFO closure in patients treated in San Raffaele Hospital

Variable	OR (95% CI)	*p*-value	C-statistics
PFO length	0.97 (0.79-1.20)	0.811	-
PFO diameter	0.87 (0.48-1.55)	0.631	-
Atrial septal aneurysm	0.62 (0.62-6.30)	0.690	-
Prominent eustachian valve	3.25 (0.54-19.38)	0.196	-
Baseline color shunt (stretched PFO)	5.80 (0.47-71.06)	0.169	-
Valsalva color shunt	5.80 (0.47-71.06)	0.169	-
Baseline positive microbubbles test*	4.50 (1.01-20.10)	**0.049**	0.675
Right-to-left shunt grade 2	1.33 (0.18-9.72)	0.777	-
Right-to-left shunt grade 3	1.20 (0.19-7.70)	0.848	-

Univariate binary logistic regression analysis for predictors of incomplete PFO closure (residual right-to-left shunt > grade 0 at 3-month TOE follow-up) with the NobleStitch EL device.
*Positive microbubbles test during normal respiration.

Table S18 - Follow-up clinical outcomes in patients treated in San Raffaele Hospital

Clinical Outcomes	Patients (N=42)
Clinical follow-up time (days)	160±34
All-cause death	0 (0)
Recurrent ischemic stroke	0 (0)
Recurrent TIA	1 (2)
Recurrent migraine*	7 (16)
Holter ECG or ILR monitoring	15 (36)
Detected arrhythmias	0 (0)
Repeated PFO closure with traditional device†	2 (4)

Data are presented as absolute numbers and percentages (for categorical variables) or mean value±SD (for continuous variables), as appropriate. Of the 65 patients included in the study, 42 (65%) underwent 3-month TOE follow-up.
*In patients with available follow-up, recurrent migraine was reported in 2 out of 6 (33%) in whom it was considered as the primary indication for PFO closure.
†Amplatzer PFO Occluder (Abbott, Santa Clara, CA, USA) was used as bailout device in all the 2 cases.

Table S19 - Bibliographic research: First Systematic Review NobleStitch EL

N	**Database**: search strategy	**Records retrieved**
1	**Medline:** NobleStitch[Title/Abstract] OR suture patent foramen ovale [Title/Abstract]	47
2	**Embase:** NobleStitch:ab,ti OR 'suture patent foramen ovale':ab,ti	8
3	**Cochrane Library:** NobleStitch: ti,ab,kw	1
4	**Centre for Reviews and Dissemination (CRD):** NobleStitch	0
5	**PROSPERO:** NobleStitch	0
6	**International Clinical Trials Registry platform (ICTRP):** NobleStitch	2
7	**ClinicalTrials.gov:** NobleStitch	2
8	**EU Clinical Trials Register:** NobleStitch	0

Date of search: June 20th, 2020

Table S20 - Bibliographic research: Second Systematic Review Amplatzer PFO Occluder

N	**Database**:search strategy	**Records retrieved**
1	**Medline:** Amplatzer PFO Occluder [Title/Abstract] AND (“Effect* closur*” [Title/Abstract] OR “Adverse effect*” [Title/Abstract])	77

Date of search: November 20th, 2020

Table S21 - Characteristics of included studies, Second Systematic Review Amplatzer PFO Occluder

N°	Ref (First Author)	Year	Journal	Country Of Implementation	Study Design	Sample Size (Tot)	Intervention	N Pts Intervention	Control	N Pts Control	Follow-Up	Follow-Up (Ecography)
1	Scalise (2)	2016	Journal of Interventional Cardiology	Italy	Case Control	205	Amplatzer PFO Occluder	52	Figulla Flex	49	53 months	TTE
2	Wahl (3)	2009	JACC: Cardiovascular Interventions	Switzerland	Cohort Study	620	Amplatzer PFO Occluder	620	/	/	6 months	TEE
3	Luani (4)	2015	EuroIntervention	Germany	Cohort Study	335	Amplatzer PFO Occluder	109	BIOstar, Cardia, Premere	68 BIOstar, 104 Cardia, 54 Premere	3 months	TEE
4	Marchese (9)	2013	EuroIntervention	Italy	Cohort Study	127	Amplatzer PFO Occluder	127	\	\		TEE
5	Meier (5)	2013	The New England journal of medicine	multicenter: Europe, Canada, Brazil, Australia	randomized clinical trial	414	Amplatzer PFO Occluder	204	medical therapy	210	4.1 years	TEE
6	Beitzke (10)	2001	Journal of Interventional Cardiology	Austria	Case Control	162	Amplatzer PFO Occluder	77	Cardioseal, Rashkind, Amplatzer ASD	73		TEE
7	Braun (6)	2003	European Hearth Journal	Germany	Case Control	307	Amplatzer PFO Occluder	69	PFO Star occluder, CardioSeal	238	21 months	TEE
8	Hammerstingl (7)	2011	European Journal Of Medical Research	Germany	Case Control	124	Amplatzer PFO Occluder	52	CardioSeal, Premere, Helex	72	87.8 month	TEE
9	Spies (11)	2008	The Journal of Invasive Cardiology	USA	Case Control	795	Amplatzer PFO Occluder	89	Cardia PFO Occluder Intrasept	Cardia PFO Occluder 405 Intrasept 301	6 months	TEE
10	Trabattoni (8)	2017	EuroIntervention	Italy	Case Control	406	Amplatzer PFO Occluder	179	Figulla	227	6 months	TTE

Table S22 - Quality Appraisal for Cohort and Case-Control studies applying New-Ottawa Scale

Cohort Studies			
**Author**	**Selection (max 4)**	**Comparability (max 2)**	**Outcome (max 3)**
Luani et al. (4)	★★★★	★★	★★★
**Case-control Studies**			
**Author**	**Selection (max 4)**	**Comparability (max 2)**	**Exposure (max 3)**
Beitzke et al. (10)	★★★★	★★	★★★
Braun et al. (6)	★★★★	★★	★★★
Scalise et al. (2)	★★★★	★★	★★★
Spies et al. (11)	★★★★	★	★★★
Trabattoni et al. (8)	★★★★	★★	★★★
Hammerstingl et al (7)	★★★★	★★	★★★

Table S23 - Quality Appraisal for the Randomized Clinical Trial study applying RoB-2 scale

Revised Cochrane risk-of-bias tool for randomized trials (RoB 2) Short Version (Cribsheet)	PP/ITT	Study Design	Randomization Process	Deviations from intended intervention	Missing outcome data	Measurement of the outcome	Selection of the reported results	Overall
Meier et al. (5)	ITT	individual RCT						

Table S24 - Amplatzer PFO Occluder safety outcome from the second systematic review

Ref (First Author)	Year	Journal	Country Of Implementation	Study Design	Sample Size (Tot)	Intervention	N Pts Intervention	Control	N Pts Control	Result On Amplatzer	Follow-Up	Ecography Used
**Procedural Time**												
Wahl (3)	2009	JACC: Cardiovascular Interventions	Switzerland	Cohort Study	620	Amplatzer PFO Occluder	620	/	/	40±21 min	6 months	TEE
Trabattoni (8)	2017	EuroIntervention	Italy	Case Control	406	Amplatzer PFO Occluder	179	Figulla	227	30.6±10.7 min	6 months	TTE
**Intraprocedural Complications**												
Scalise (2)	2016	Journal of Interventional Cardiology	Italy	Case Control	205	Amplatzer PFO Occluder	52	Figulla Flex	49	0%	53 months	TTE
Wahl (3)	2009	JACC: Cardiovascular Interventions	Switzerland	Cohort Study	620	Amplatzer PFO Occluder	620	/	/	0.8%	6 months	TEE
Meier (5)	2013	The New England journal of medicine	Europe, Canada, Brazil, Australia	multicenter randomized clinical trial	414	Amplatzer PFO Occluder	204	medical therapy	210	1.5%	4.1 years	TEE
Trabattoni (8)	2017	EuroIntervention	Italy	Case Control	406	Amplatzer PFO Occluder	179	Figulla	227	5%	12 months	TTE
**Atrial Fibrillation**												
Scalise (2)	2016	Journal of Interventional Cardiology	Italy	Case Control	205	Amplatzer PFO Occluder	52	Figulla Flex	49	3.8%	53 months	TTE
Beitzke (10)	2001	Journal of Interventional Cardiology	Austria	single center, observational study	162	Amplatzer PFO Occluder	77	Cardioseal, Rashkind, Amplatzer ASD	73	3.9%		TEE
Hammerstingl (7)	2011	European Journal of Medical Research	Germany	Case Control	124	Amplatzer PFO Occluder	52	CardioSeal, Premere, Helex	72	1.9%	87.8 month	TEE
Spies (11)	2008	The Journal of Invasive Cardiology	USA	Case Control	795	Amplatzer PFO Occluder	89	Cardia PFO Occluder Intrasept	Cardia PFO Occluder 405 Intrasept 301	10.1%	6 months	TEE

Luani (4)	2015	EuroIntervention	Germany	Cohort Study	335	Amplatzer PFO Occluder	109	BIOstar, Cardia, Premere	226	2%	5 years	TEE
Trabattoni (8)	2017	EuroIntervention	Italy	Case Control	406	Amplatzer PFO Occluder	179	Figulla	227	17% Supraventricular arrhythmias 2.2% Paroxysmal atrial fibrillation	6 months	TTE
**Device-Related Complications**												
Wahl (3)	2009	JACC: Cardiovascular Interventions	Switzerland	Cohort Study	620	Amplatzer PFO Occluder	620	/	/	1.3% second device implantation	6 months	TEE
Wahl (3)	2009	JACC: Cardiovascular Interventions	Switzerland	Cohort Study	620	Amplatzer PFO Occluder	620	/	/	0.5% thrombus identification	6 months	TEE
Luani (4)	2015	EuroIntervention	Germany	Cohort Study	335	Amplatzer PFO Occluder	109	BIOstar, Cardia, Premere	226	2% TIA	4.1 years	TEE
Meier (5)	2013	The New England journal of medicine	Europe, Canada, Brazil, Australia	multicenter randomized clinical trial	414	Amplatzer PFO Occluder	204	medical therapy	210	1% death 0.5% non-fatal stroke 2.5% TIA	6 months to 5 years	TEE
Braun (6)	2003	European Hearth Journal	Germany	Observational study	307	Amplatzer PFO Occluder	69	PFO Star occluder, CardioSeal	238	1.4% Periferic Emboli	21 months	TEE
Hammerstingl (7)	2011	European Journal of Medical Research	Germany	Case Control	124	Amplatzer PFO Occluder	52	CardioSeal, Premere, Helex	72	3.8% Pericardial Effusion, Embolization	87.8 months	TEE
Trabattoni (8)	2017	EuroIntervention	Italy	Case Control	406	Amplatzer PFO Occluder	179	Figulla	227	0% Death, Ischaemic stroke, TIA, Aortic erosion	12 months	TTE
Spies (11)	2008	The Journal of Invasive Cardiology	USA	Case Control	795	Amplatzer PFO Occluder	89	Cardia PFO Occluder Intrasept	Cardia PFO Occluder 405 Intrasept 301	1.1% thrombus formation 1.1% TIA 1.1% stroke	6 months	TEE

Table S25 - Profiles of Professionals interviewed for Qualitative Domains

Number	Profile	Institution
6	Experienced clinicians<br>• 2 Cardiologists<br>• 2 Cardiologists or Anesthesiologists<br>• 2 Cardiovascular imaging experts	San Raffaele Hospital
1	Head of Interventional Cardiology Department;	
1	Head nurse of Interventional Cardiology Department;	
1	Head of Cardiovascular Imaging Department;	
1	Head of Cardiac Surgery Department.	
Total 10		

Bibliography

1. Gaspardone A, D.M.F., Sgueglia GA, et al. Novel percutaneous suture-mediated patent foramen ovale closure technique: early results of the NobleStitch EL Italian Registry. EuroIntervention. 2018;14(3):e272-e279. Published 2018 Jun 8. doi:10.4244/EIJ-D-18-00023.

2. Scalise F, Auguadro C, Sorropago G, Sorropago A, Novelli E, Finizio M, et al. Long-Term Contrast Echocardiography and Clinical Follow-Up after Percutaneous Closure of Patent Foramen Ovale Using Two Different Atrial Septal Occluder Devices. J Intervent Cardiol. agosto 2016;29(4):406–13.

3. Wahl A, Tai T, Praz F, Schwerzmann M, Seiler C, Nedeltchev K, et al. Late results after percutaneous closure of patent foramen ovale for secondary prevention of paradoxical embolism using the amplatzer PFO occluder without intraprocedural echocardiography: effect of device size. JACC Cardiovasc Interv. febbraio 2009;2(2):116–23.

4. Luani B, Markovic S, Krumsdorf U, Rottbauer W, Wöhrle J. Efficacy of different devices for transcatheter closure of patent foramen ovale assessed by serial transoesophageal echocardiography and rates of recurrent cerebrovascular events in a long-term follow-up. EuroIntervention J Eur Collab Work Group Interv Cardiol Eur Soc Cardiol. maggio 2015;11(1):85–91.

5. Meier B, Kalesan B, Mattle HP, Khattab AA, Hildick-Smith D, Dudek D, et al. Percutaneous closure of patent foramen ovale in cryptogenic embolism. N Engl J Med. 21 marzo 2013;368(12):1083–91.

6. Braun M, Gliech V, Boscheri A, et al. Transcatheter closure of patent foramen ovale (PFO) in patients with paradoxical embolism. Periprocedural safety and mid-term follow-up results of three different device occluder systems. Eur. Heart J. 2004;25:424–30.

7. Hammerstingl C, Bauriedel B, Stüsser C, Momcilovic D, Tuleta I, Nickenig G, et al. Risk and fate of residual interatrial shunting after transcatheter closure of patent foramen ovale: a long term follow up study. Eur J Med Res. 27 gennaio 2011;16(1):13–9.

8. Trabattoni D, Gaspardone A, Sgueglia GA, Fabbiocchi F, Gioffrè G, Montorsi P, et al. AMPLATZER versus Figulla occluder for transcatheter patent foramen ovale closure. EuroIntervention J Eur Collab Work Group Interv Cardiol Eur Soc Cardiol. 20 aprile 2017;12(17):2092–9.

9. Marchese N, Pacilli MA, Inchingolo V, Fanelli R, Loperfido F, Vigna C. Residual shunt after percutaneous closure of patent foramen ovale with AMPLATZER occluder devices - influence of anatomic features: a transcranial Doppler and intracardiac echocardiography study. EuroIntervention J Eur Collab Work Group Interv Cardiol Eur Soc Cardiol. luglio 2013;9(3):382–8.

10. Beitzke A, Schuchlenz H, Gamillscheg A, Stein JI, Wendelin G. Catheter closure of the persistent foramen ovale: mid-term results in 162 patients. J Intervent Cardiol. aprile 2001;14(2):223–9.

11. Spies C, Reissmann U, Timmermanns I, Schräder R. Comparison of contemporary devices used for transcatheter patent foramen ovale closure. J Invasive Cardiol. settembre 2008;20(9):442–7.
